# Transformative Effect of COVID-19 Pandemic on Magnetic Resonance Imaging Services in One Tertiary Cardiovascular Center

**DOI:** 10.3390/jimaging9060108

**Published:** 2023-05-28

**Authors:** Tatiana A. Shelkovnikova, Aleksandra S. Maksimova, Nadezhda I. Ryumshina, Olga V. Mochula, Valery K. Vaizov, Wladimir Y. Ussov, Nina D. Anfinogenova

**Affiliations:** 1 Cardiology Research Institute, Branch of the Federal State Budgetary Scientific Institution "Tomsk National Research Medical Center of the Russian Academy of Sciences", 634012 Tomsk, Russia; 2 Meshalkin National Medical Research Center, 630055 Novosibirsk, Russia

**Keywords:** COVID-19, long COVID-19, paramagnetic contrast-enhanced cardiac magnetic resonance imaging, MRI, CMR, hypertrophic cardiomyopathy, myocardial fibrosis, myocarditis, electronic medical record

## Abstract

The aim of study was to investigate the transformative effect of the COVID-19 pandemic on magnetic resonance imaging (MRI) services in one tertiary cardiovascular center. The retrospective observational cohort study analyzed data of MRI studies (*n* = 8137) performed from 1 January 2019 to 1 June 2022. A total of 987 patients underwent contrast-enhanced cardiac MRI (CE-CMR). Referrals, clinical characteristics, diagnosis, gender, age, past COVID-19, MRI study protocols, and MRI data were analyzed. The annual absolute numbers and rates of CE-CMR procedures in our center significantly increased from 2019 to 2022 (*p*-value < 0.05). The increasing temporal trends were observed in hypertrophic cardiomyopathy (HCMP) and myocardial fibrosis (*p*-value < 0.05). The CE-CMR findings of myocarditis, acute myocardial infarction, ischemic cardiomyopathy, HCMP, postinfarction cardiosclerosis, and focal myocardial fibrosis prevailed in men compared with the corresponding values in women during the pandemic (*p*-value < 0.05). The frequency of myocardial fibrosis occurrence increased from ~67% in 2019 to ~84% in 2022 (*p*-value < 0.05). The COVID-19 pandemic increased the need for MRI and CE-CMR. Patients with a history of COVID-19 had persistent and newly occurring symptoms of myocardial damage, suggesting chronic cardiac involvement consistent with long COVID-19 requiring continuous follow-up.

## 1. Introduction

The 2019 novel coronavirus infection (COVID-19) changed the profile of patients seeking primary medical care. Concomitant injury to the lungs and myocardium in COVID-19 was observed using magnetic resonance imaging (MRI) at the very beginning of the COVID-19 pandemic [1,2,3,4]. Recent data showed that the number of patients with heart problems indeed significantly increased during the COVID-19 pandemic [5,6,7,8]. Most patients with the symptoms of heart disease reported recent respiratory illness and confirmed COVID-19 infection caused by SARS-CoV-2 virus. Numerous studies were performed focusing on the possible mechanisms of damage to the cardiovascular system after COVID-19 [6,9,10]. However, precise pathophysiological mechanisms remain poorly understood.

From the very beginning of the COVID-19 pandemic, magnetic resonance imaging (MRI) data suggested that COVID-19-associated damage occurred due to cellular and circulating factors present not only in the pulmonary parenchyma itself, but also in the vascular wall, myocardium [11,12], and brain tissue [13]. An increasing number of patients have been suffering from persisting symptoms even months after COVID-19 [2,5,14], although the availability of effective vaccines resulted in a decrease in the burden of acute coronavirus infection [15,16].

In regard to cardiac health, diagnostic algorithms for COVID-19 typically aim at ruling out the inflammatory changes in the myocardium. At the same time, the incidence of other nosological entities, in particular cardiomyopathy, progressively increases. Contrast-enhanced cardiac MRI (CE-CMR) is considered the method of choice for instrumental diagnostics of cardiological pathology according to the recommendations of the European Society of Cardiology and American Heart Association [17,18], and the Society for Cardiovascular Magnetic Resonance (SCMR) recommended CMR protocols for scanning patients with active or convalescent-phase COVID-19 infection [1]. Our previous data showed that CE-CMR allows detecting cardiac involvement in about 37% of patients with tomography-documented lung injury, and the vast majority of these patients have multiple focal lesions in the myocardium [13]. Considering that SARS-CoV-2 virulence and clinical manifestation of COVID-19 are fluctuating with time, continuous monitoring and the development of effective strategy and tactics for administering the diagnostic procedures are required. 

On the other hand, little is known about the effect of the COVID-19 pandemic on the patterns of administration of MRI and other medical imaging modalities, while the use of these methods represents an essential resource for timely diagnosis and continuing monitoring of acute and chronic COVID-19. It is essential to understand the impact of the COVID-19 pandemic on the cardiac health of the population and the ways to timely diagnose and control the disease because the majority of the population suffered from confirmed or unconfirmed COVID-19 infection with potential chronic complications, which may significantly differ from those of ordinary acute respiratory diseases and be severe. 

The aim of the study was to gain insight into the transformative effect of the COVID-19 pandemic on MRI administration during the COVID-19 pandemic, with emphasis on COVID-19’s impact on cardiac health. 

## 2. Materials and Methods

### 2.1. Sample Characteristics

The retrospective observational cohort study was performed in accordance with the standards of Good Clinical Practice and the Declaration of Helsinki. 

The study aimed to analyze data of MRI studies (*n* = 8137) performed in one tertiary cardiovascular center from 1 January 2019 to 1 June 2022. The CE-CMR examinations comprised 12.13% of these studies. The CE-CMR sample (*n* = 987) included the patients aged 0 to 89 years who had indications for CE-CMR study and underwent a CE-CMR procedure within the above-mentioned period. Contraindications for CE-CMR study were high risk of adverse reactions to paramagnetic contrast enhancement, implantation of artificial pacemaker, ferromagnetic clips in the brain and spinal cord, the presence of any foreign object partially or completely within the orbit, with or without penetration or perforation of the globe, implanted automatic drug dispensers, severe heart rhythm disorder, large joint prosthesis, the first trimester of pregnancy, and claustrophobia. 

Patient sex was identified according to electronic health record data based on patient ID documents presented for establishing the medical chart. Cardiovascular diagnosis of patients was established and/or verified by a cardiologist.

### 2.2. MRI Data Analysis

Medical records with the results of CE-CMR examinations were acquired from the electronic module, previously designed to store data of instrumental studies. Data contained standard information of the CE-CMR report including patient demographic and clinical data, characteristics of the CE-CMR procedure, suspected diagnosis, verified diagnosis, and type of examination (inpatient or outpatient). Patients received a standard diagnostic CE-CMR procedure using a 1.5-tesla MRI system Vantage Titan 1.5T (Toshiba Medical Systems Corporation, Otawara-Shi, Tochigi-ken, Japan) with electrocardiogram and breathing synchronization and myocardial image acquisition along the short and long axes before and after administration of gadolinium-based contrast agent gadobutrol (Gadovist, Gadobuskan). The thickness of slices was 5–7 mm and the images were recorded into a 256 × 256 matrix. The protocol of the CE-CMR study included T1- and T2-weighted sequences with fat tissue signal suppression for the evaluation of myocardial condition, dynamic SSFP-sequences for assessment of the volumes and function of the left ventricle, and gradient inversion recovery sequences (GR-IR) for the detection of areas with pathologic contrasting. Inversion time was adjusted individually (on average, TI = 300 ± 10 ms). The evaluation of affected areas in the myocardium was carried out using a 17-segment system of topical characterization of the left ventricular myocardium. Referrals, clinical characteristics, diagnosis, gender, age, type of visit, MRI study protocols, and MRI data were analyzed. 

### 2.3. Sample Size Calculation

The sufficiency of the sample size was established based on the following consideration: we considered an acceptable margin of error of 5%, confidence level of 95%, and approximate population size of 20,000. Taking into account an assumed 50%-response distribution, we calculated a sufficient sample size of 377 patients as follows: the sample size *n* and margin of error *E* are given by

x=Z×(c100)2×r(100−r)

n=N×x((N−1)×E2+x)

E=(N−n)×xn×(N−1)

where *N* is the population size, *r* is the fraction of responses that we are interested in, Z is the critical value for the confidence level *c*, and *x* is the coefficient based on critical value for confidence level and fraction of responses used to facilitate calculation of *n* and *E*. Taking into account that the sample of patients who underwent the CE-CMR procedure exceeded the calculated sample size, we considered the size sufficient. 

### 2.4. Statistical Processing of Data

The normality of distribution of variables was checked with the Kolmogorov–Smirnov test and the Shapiro–Wilk test. Data are presented as percentages and absolute numbers. Categorical variables were compared with Pearson’s chi-square test using multifield contingency tables. Values were considered statistically significant when the *p*-value was <0.05.

### 2.5. Study Support

The study was supported by the Russian Science Foundation (grant #22-I5-00313) with regard to study design, data acquisition, data analysis, data interpretation, and publication of the research results. The founding agency did not influence in any way the integrity of the data presented. 

## 3. Results

### 3.1. Temporal Trend in CE-CMR Procedures

**Figure 1:**
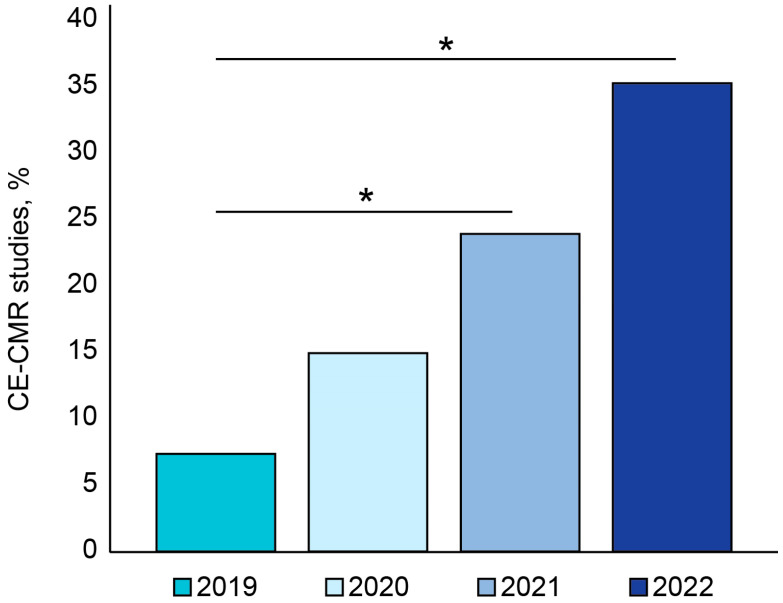
Percentage of CE-CMR studies among all MRI studies in one tertiary cardiovascular center year-by-year. * *p*-value < 0.05, CE-CMR: contrast-enhanced cardiac MRI.

The year-by-year dynamics in the number of CE-CMR procedures (*n* = 987) in our study was as follows: 198 studies (20.1%) were performed in 2019; 232 studies (23.5%) were conducted in 2020 (*p*-value < 0.05); 330 examinations (33.4%) were performed in 2021 (*p*-value < 0.05); and 227 studies (23%) were conducted within the first six months of 2022 (*p*-value < 0.05). Besides a significant increase in the absolute number of CE-CMR procedures, the annual proportion of these studies increased and continued to increase among the total number of MRI exams (*p* < 0.05) (Figure 1). The number of outpatients was 32 (16%) in 2019, 68 (29%) in 2020, 57 (17%) in 2021, and 40 (18%) during the first six months of 2022, suggesting significant increases in 2020 and 2022 relative to 2019 (*p*-value < 0.05). 

### 3.2. Sex-Related Differences in CE-CMR-Detected Cardiac Pathologies during COVID-19 Pandemic

The rates of male cardiac patients who received CE-CMR examinations were 58.59% in 2019, 51.72% in 2020, and 56.36% in 2021, and the rate significantly increased to 73.13% during the first six months of 2022 *(p* < 0.05). A similar temporal increase in the rates of CE-CMR examination studies to 2022 was observed in women *(p* < 0.05). The numbers of cases with fibrotic–dystrophic changes in the myocardium significantly increased and continued to increase to 2022 in both male (from 52.57% in 2019 to 81.03% in 2022, *p* < 0.05) and female patients (from 62.20% in 2019 to 87.39% in 2022, *p* < 0.05). The proportion of cases with detected acute myocarditis decreased to 10% in male and to 13% in female patients in 2020 among all detected cardiac pathologies, with a subsequent gradual increase in 2021. 

**Figure 2:**
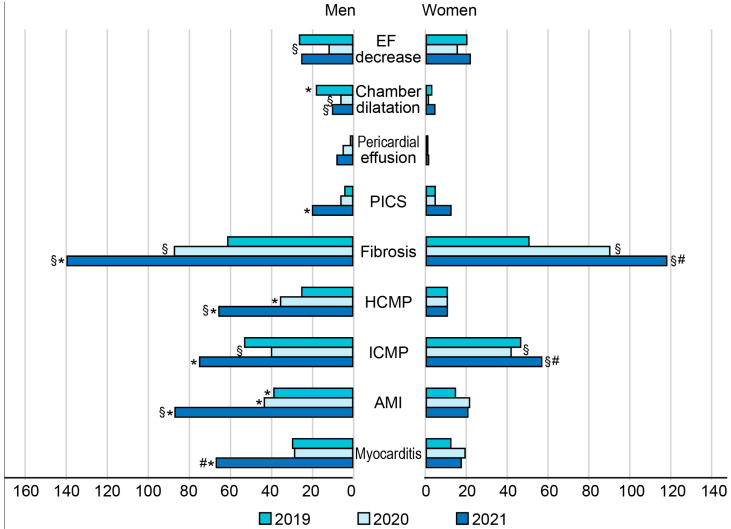
Distribution of cardiac pathologies in male and female patients who underwent CE-CMR studies in one tertiary cardiovascular center during COVID-19 pandemic. AMI—acute myocardial infarction, EF—ejection fraction, ICMP—ischemic cardiomyopathy, HCMP—hypertrophic cardiomyopathy, PICS—postinfarction cardiosclerosis. Horizontal axis corresponds to the number of patients. * *p*-value < 0.05 for sex-related difference. ^§^ *p*-value < 0.05 for significant difference in the incidence compared with the pre-pandemic year (2019). ^#^ *p*-value < 0.05 for significant difference in the incidence compared with the previous year (2021 vs. 2020).

A sex-related comparison in our cohort showed a significantly higher CE-CMR-based incidence of acute myocardial infarction (AMI), hypertrophic cardiomyopathy (HCMP), ischemic cardiomyopathy (ICMP), postinfarction cardiosclerosis (PICS), myocardial fibrosis, and myocarditis in men compared with the corresponding numbers in women in 2021 (*p* < 0.05) (Figure 2). Interestingly, while the year-by-year occurrence of HCMP increased in men (*p* < 0.05), this parameter remained stable, showing no temporal trend in women in 2019–2021. 

### 3.3. Age-Related Distribution of Patients Who Underwent CE-CMR Study during COVID-19 Pandemic

**Figure 3:**
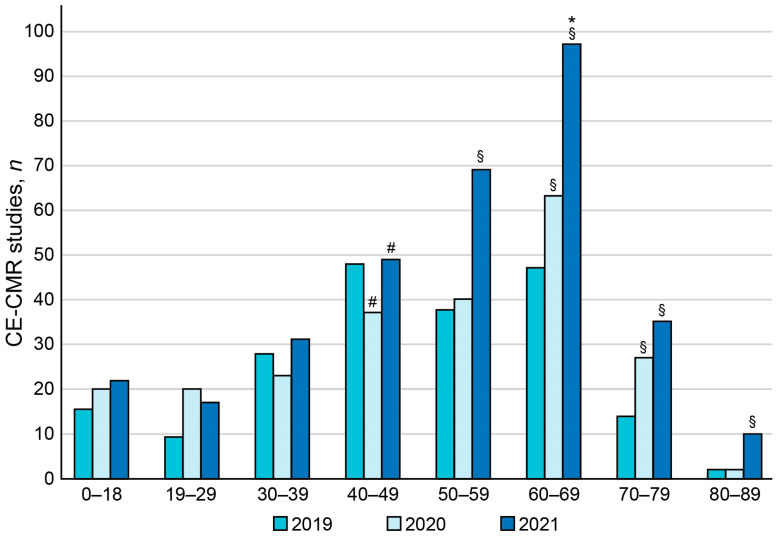
Age distribution of patients who received CE-CMR studies in one tertiary cardiovascular center during COVID-19 pandemic. Vertical axis corresponds to the number of patients. Horizontal axis corresponds to the age groups. ^§^ *p*-value < 0.05 for significant difference in absolute number compared with the pre-pandemic year (2019). ^#^ *p*-value < 0.05 for significant difference in the incidence compared with the previous year. * *p*-value < 0.05 for the highest increment in the number compared with other age groups.

The age-related analysis showed that the peak in the number of patients in each age group was in 2021, and the frequency of occurrence of cardiac pathologies was the highest in 60–69-year-old patients with a high risk for a severe course and complications of COVID-19 (*p*-value < 0.05). Among the total amount of CE-CMR studies, patients aged 60–69 years comprised 11.90% in 2019, 15.95% in 2020, 24.56% in 2021 (*p*-value < 0.05 vs. 2019), and 18.73% in 2022 (Figure 3). The age groups of 60–69- and 70–79-year-olds showed the most consistent increasing temporal trends in the number of administered CE-CMR procedures in 2019–2021. The highest increment in the annual number of CE-CMR studies was observed in group of 60–69-year-old patients (*p*-value < 0.05).

### 3.4. Year-by-Year Distribution of CE-CMR-Detected Cardiac Pathologies during COVID-19 Pandemic

**Figure 4:**
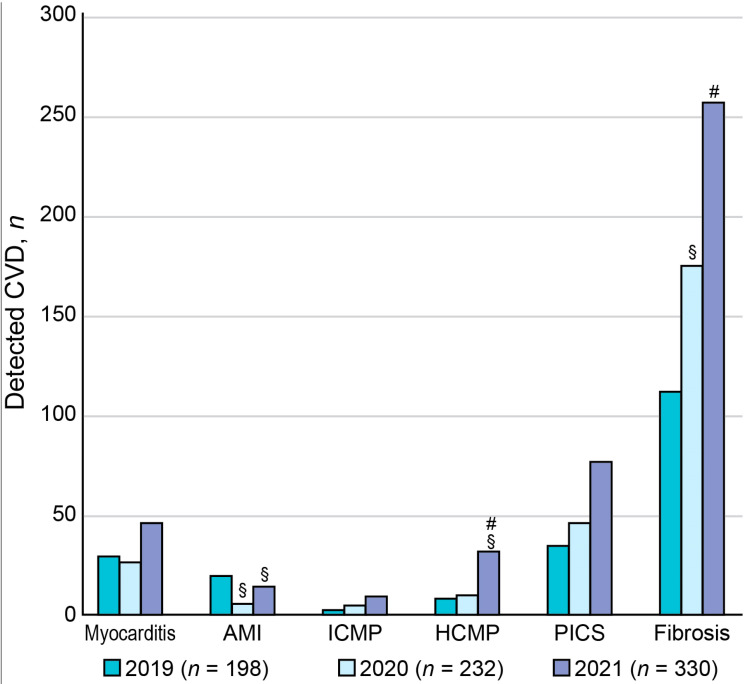
Distribution of cardiovascular diseases in patients who received CE-CMR studies in one tertiary cardiovascular center during COVID-19 pandemic. ^§^ *p*-value < 0.05 compared with the incidence in the pre-pandemic year (2019). ^#^ *p*-value < 0.05 compared with the incidence in the previous year (2021 vs. 2020).

**Figure 5:**
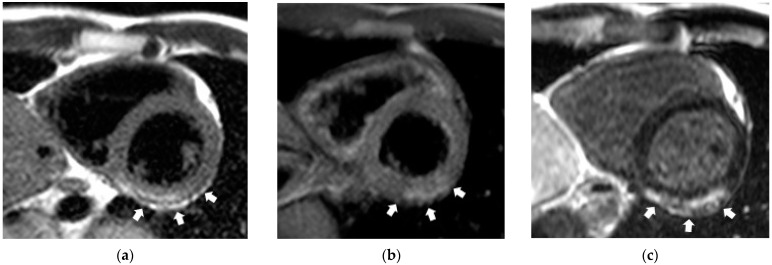
Contrast-enhanced cardiac magnetic resonance imaging in a patient with history of COVID-19 and three Lake Louise criteria of myocardial inflammation: edema, hyperemia, and fibrosis. White arrows indicate (**a**) edema (T2-weighted image), (**b**) hyperemia of the left ventricular inferior wall (early post-contrast enhancement on T1-weighted image), and (**c**) fibrosis (late contrast enhancement in inversion recovery mode).

The main diagnostic entities of cardiovascular pathology, detected using CE-CMR, were myocarditis, AMI, ICMP, HCMP, PICS, and myocardial fibrosis (Figure 4). Myocardial fibrosis was characterized by the presence of non-coronarogenic fibrotic foci (Figure 5). 

The analysis of the year-by-year distribution of these cardiac pathologies, except for AMI, showed that the incidence of each detected entity peaked in 2021 (*p*-value < 0.05). Moreover, the proportion of patients with myocardial fibrosis, which could be a consequence of myocardial inflammation, significantly increased among the entire cohort of patients who received CE-CMR study: 67.17% in 2019, 76.29% in 2020, 78.18% in 2021, and 84.14% before June 2022 (*p* < 0.05) (Figure 4). The consistent increasing temporal trends occurred for HCMP, myocarditis, and myocardial fibrosis (*p* < 0.05). 

### 3.5. CE-CMR-Detected Non-Coronarogenic Myocardial Fibrosis during COVID-19 Pandemic

**Figure 6:**
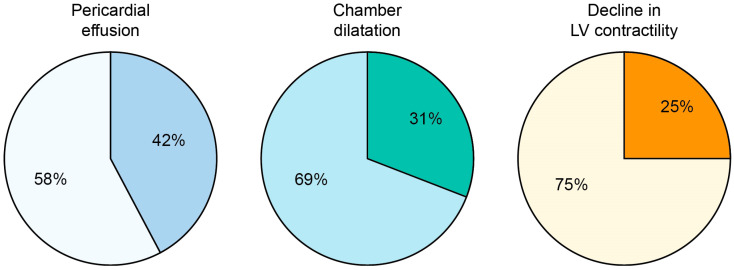
Pericardial effusion, cardiac chamber dilatation, and decline in the left ventricular (VL) contractility in the group of patients with fibrotic dystrophy changes. Darker sectors correspond to the proportion of patients who had these changes.

The CE-CMR studies in patients with non-coronarogenic myocardial fibrosis showed the presence of pericardial effusion in 42% of patients, dilatation of cardiac chambers in 31% of patients, and a decrease in the left ventricular contractile function in 25% of patients (Figure 6). 

### 3.6. Temporal Trends in CE-CMR Administrations and COVID-19 Occurrence during the Pandemic

**Figure 7:**
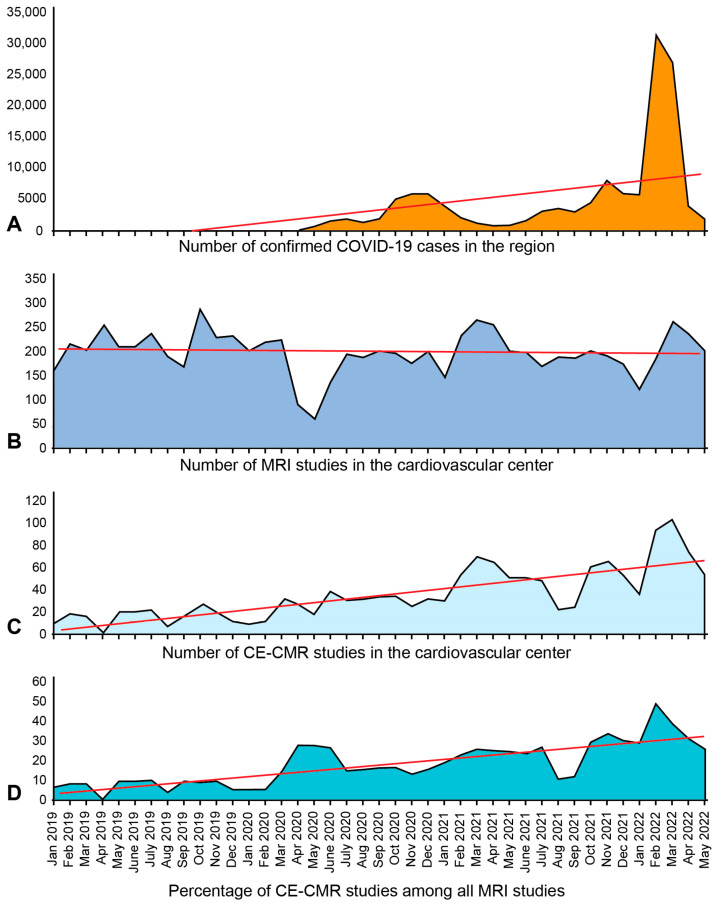
Trends in diagnostic imaging procedures during COVID-19 pandemic. (**A**) Number of confirmed COVID-19 cases in the region month-by-month. (**B**) Number of MRI studies performed during COVID-19 pandemic in one tertiary cardiovascular center. (**C**) Number of CE-CMR studies. (**D**) Percentage of CE-CMR studies among all MRI studies. Red lines indicate the corresponding linear trends.

The COVID-19 incidence was characterized by wave-like behavior with an increasing trend at the population level in 2020–2022. The first wave of coronavirus infection had the most negative impact on a patient stream in our institute (April to May 2020) when there was a dramatic drop in the total number of performed studies in May 2020 (*p* < 0.001), perhaps due to restrictive measures, self-isolation, a decline in pre-arranged hospital care, etc. (Figure 7).

## 4. Discussion

We performed a retrospective study of an MRI database [19] to elucidate the transformative effect of the COVID-19 pandemic on magnetic resonance imaging services in one tertiary cardiovascular center. Our study allowed us to establish the temporal and sex-related patterns of MRI findings in a single cardiovascular center. 

The annual absolute numbers and rates of CE-CMR procedures in our center significantly increased from 2019 to 2022. The proportions of CE-CMR studies performed on an outpatient and inpatient basis showed the oscillations during this period due to the implementation of restrictive measures preventing the spread of COVID-19 infection. A pronounced year-by-year increase in the detection of fibrotic–dystrophic changes in the myocardium was a remarkable observation in our cohort of patients, and this observation was associated with the presence of non-coronarogenic fibrotic foci. The annual detection rates of AMI did not show any consistent dynamics, which may be explained by the restrictive measures preventing patients with acute COVID-19 infection from visiting our center so that we dealt mostly with the manifestation of long COVID-19, while acute conditions were treated in respiratory hospitals. The consistent increasing temporal trends occurred for HCMP, myocarditis, and focal myocardial fibrosis. Interestingly, the CE-CMR findings of myocarditis, AMI, HCMP, PICS, and focal myocardial fibrosis prevailed in men compared with those in women at the time of the pandemic. Moreover, while the occurrence of HCMP significantly increased with time in men, this parameter remained stable in women. Our data showed that, expectedly, the older patients represented the most vulnerable category. The study revealed the consistence between the temporal trends in CE-CMR administrations and COVID-19 occurrence during the pandemic. While the month-by-month numbers of overall MRI studies remained relatively stable, except for isolated fluctuations such as in May 2020, the absolute numbers and the proportion of CE-CMR studies showed consistent increasing trends (*p* < 0.001) concordant with those of COVID-19 incidence.

The global COVID-19 pandemic put unprecedented pressure on the healthcare system worldwide. The first wave of this viral infection was associated with a decline in most areas of healthcare such as planned and screening procedures, including examinations requiring MRI [20,21,22,23]. It is not surprising that a significant drop in the number of diagnostic MRI procedures was observed in our cardiovascular center during the first months of the COVID-19 pandemic. 

The analysis of MRI studies performed in 2019–2022 showed a significant increase in the number of myocardial imaging procedures. This observation agrees well with data of world statistics and confirms the need in cardiac exams considering, to a lesser degree, the direct and largely indirect (through systemic inflammatory reaction) impact of SARS-CoV-2 on the cardiovascular system. Moreover, cardiovascular findings could be associated with the side effects of drugs administered for COVID-19 treatment, especially when antiviral medications are used. In this regard, potential drug–drug interactions between antiviral drugs, oral anticoagulants, and other medications for the treatment of COVID-19 may represent an essential and poorly studied problem [2,24,25]. 

Our study confirmed the effect of patient age and sex on the risk of developing cardiovascular complications, which also agrees with data from the literature [26]. The age-related analysis showed that cardiovascular pathology was significantly more often detected in older patients. 

The CE-CMR-based assessment of inflammatory changes in the myocardium was performed in our center using the Lake Louise Criteria [27], which specify three aspects of myocardial inflammation: edema, hyperemia, and necrosis and/or fibrosis based on an assessment of signal intensity on T2-weighted images, in regimes of early and delayed contrast enhancement of the myocardium. These criteria were identified based on a limited number of published studies, and, at that time, their diagnostic accuracy, sensitivity, and specificity were estimated to be 78%, 67%, and 91%, respectively. Though some criteria and approaches to the assessment have been a matter of debate, these numbers were largely confirmed and validated by later studies [28]. The use of CE-CMR signal intensity for the detection of myocardial edema has some disadvantages. When the inflammation spreads, which usually happens when myocarditis transits from acute to subacute stage, the intensity of T2-weighted images and contrasting also becomes more uniform, to the degree that individual lesions can no longer be easily detected by qualitative review. 

In our study, the CE-CMR-based signs of acute inflammation in the myocardium were present only in a quarter of patients who underwent CE-CMR study in 2019, with a gradual decrease in the proportion of such patients to 9.4% in 2022, though the majority of patients who recovered from COVID-19 reported cardiac symptoms. This phenomenon may originate from two causes. First, the chest symptoms could be related to the residual pulmonary disease, but this hypothesis requires further investigation. Second, considering that the average time between the occurrence of the first symptoms and CE-CMR examination ranged from one to two months, the studies were likely performed during edema regression at the subacute stage. The recently proposed new CMR modalities include T1- and T2-relaxation time mapping and determination of extracellular volume fraction that could potentially expand the diagnostic capabilities of CE-CMR in patients with myocarditis [29]. The T1- and T2-mapping allows quantitatively assessing the diffuse changes such as diffuse edema and fibrosis in the myocardium. Unfortunately, these methods of mapping have been unavailable in our center. 

The small foci of myocardial fibrosis of non-coronarogenic genesis were detected in the vast majority of patients who received CE-CMR in our center, which agrees with our previously published works [12,13]. The presence of pericardial effusion and/or abnormal contractile function of the left ventricle was observed in some of our patients in this group (Figure 7). The detected changes may result from past inflammatory processes triggering cardiac remodeling. Indeed, the literature suggests that myocardial remodeling may precede the functional left ventricular remodeling [30]. Our results are in compliance with this conclusion as the abnormalities were detected mostly in the left myocardial structure rather than function in the majority of subjects. Therefore, the patients of this group require a long-term follow up with repeated CE-CMR as a sensitive tool for monitoring the progress of the recovery. 

It is worth emphasizing that we observed an increase in the incidence of other cardiovascular pathology including ICMP and HCMP during the COVID-19 pandemic, which can be caused by both the direct effects of complications of SARS-CoV-2 infection and the side effects of pharmacological treatment in cardiovascular patients with COVID-19 [24]. The latter issue remains poorly understood and requires further study as the pharmacokinetics of antiviral agents such as atazanavir, lopinavir, and ritonavir interfere with the metabolism of certain cardiovascular medications, including antiarrhythmics and anticoagulants. It potentially leads to cardiac injury or asystole due to QTc time prolongation or bleeding [31], which is essential for the group of cardiovascular patients. 

We found increases in the rates of diagnostic procedures and detected pathological changes in the cardiovascular system during the third year of the COVID-19 pandemic, which also may suggest a chronic course of this disease. Prospects for the wider use of MRI in diagnosing not only chronic cardiac but also chronic lung complications of COVID-19 require further exploration, considering that pilot data show MRI to be an accurate and informative imaging modality in this regard [12,32,33,34,35,36]. The CMR may be considered the most useful noninvasive test to detect myocarditis and can give detailed information in terms of myocardial structure and function [37,38,39]. The CMR findings of patchy myocardial edema in high-school athletes with normal echocardiograms and cardiac biomarkers suggest that all symptomatic patients of all ages should undergo additional cardiac testing, including CE-CMR [40]. Modified scanning protocols during the COVID-19 pandemic may reduce the risk of exposure while providing essential data regarding cardiac tissue inflammation, fibrosis, cardiac remodeling, and contractile function. The growing use of CMR in clinical practice to assess myocardial injury will improve understanding of the acute and chronic sequelae of myocardial inflammation from various pathological etiologies [39]. 

A comparison of our findings with the results obtained in other studies suggests that the impact of the COVID-19 pandemic on cardiac health in our region was relatively severe, considering that our patients had a history of only mild-to-moderate COVID-19. For example, the incidence rate of acute myocarditis diagnosed according to Lake Louise MRI criteria in our center (9.4% in 2022) exceeded the corresponding incidence rate (6.7%) in hospitalized patients with elevated troponin levels and COVID-19 across 25 hospitals in the United Kingdom [41]. An international cohort study enrolling 100 patients from Germany, with every third having a severe disease requiring hospitalization, showed that 20% of these patients had a nonischemic late gadolinium enhancement pattern within 10 weeks after positive COVID-19 testing [42]. In our study, the incidence rate of nonischemic late contrast enhancement in inversion recovery mode (suggesting the presence of fibrosis) reached ~84% in 2022. Seidel et al. reported no evidence of myocardial inflammation, fibrosis, or functional cardiac impairment in children who recovered from mildly symptomatic COVID-19 infections between November 2020 and January 2021 [43]. In our study, we observed the signs of cardiac involvement in the group of patients aged 0 to 18 years with a history of mild-to-moderate COVID-19, but the highest incidence of cardiac involvement was expectedly found in older adults. The observed differences between the incidence rates may be explained by the variations in the cardiotropic virulence of SARS-CoV-2 strains in different countries [44,45], the geographic specifics of appropriate use criteria underlying referrals to imaging studies, and the diversity of patient populations.

The limitation of our study was the impossibility of using the MRI mapping protocols due to the limited capabilities of equipment available in our center. Moreover, most of the enrolled patients who received CE-CMR had a history of mild or moderate COVID-19, so our report does not cover the entire spectrum of manifestations of this pathology because we did not study critically ill patients or those who suffered from severe COVID-19. The proportion of patients with cardiac involvement detected in our study may not be extrapolated to a wider population and other patient cohorts. Nevertheless, the present study demonstrates the phenomenon of cardiac involvement after COVID-19, and the obtained results may be useful because they contribute to developing the awareness of medical specialists. It is all the more essential considering that under-diagnosis of cardiac lesions most likely occurs in patients with a mild form of SARS-CoV-2 infection. Our data agree with the observation that mild SARS-CoV-2 infection leaves long-lasting effects on cardiovascular health [5]. At last, we completed the cross-sectional observational electronic medical-record-based MRI investigation, whereas prolonged observation would be helpful for the evaluation of myocardial condition over time, especially considering the risk of developing chronic cardiac pathology in patients with past history of mild and moderate COVID-19.

## 5. Conclusions

The COVID-19 pandemic had a transformative effect on magnetic resonance imaging services in the tertiary cardiovascular center and was associated with an increased demand in CE-CMR. The observed pattern of cardiac involvement in individuals with past history of COVID-19 suggests that patients with cardiac symptoms require continuous follow up to prevent potential pathological cardiac remodeling. CE-CMR evaluation should be considered in patients with acute COVID-19 infection, long COVID-19, and during the recovery period to detect cardiac injury and provide risk stratification as early as possible. Educational and organizational efforts are needed to promote the use of CE-CMR and other relevant imaging modalities among populations affected by the COVID-19 pandemic. An increase in affordability and availability of medical imaging is strategically essential for population health. 

## Data Availability

Deidentified individual participant data (text, tables, figures, and appendices), underlying the results of the trial, will be shared with researchers to achieve the aims in the approved proposal. Time Frame: Proposals may be submitted up to 36 months following publication of the results of the trial. After 36 months, the data will be available in the center’s data warehouse but without investigator support other than deposited metadata. Access Criteria: Information regarding submitting proposals and accessing data may be requested from the corresponding author by e-mail.
